# Blinking-Based Identification of Single Dye Molecules in Ink

**DOI:** 10.1021/acs.analchem.5c06137

**Published:** 2026-01-02

**Authors:** Alisha J. Khodabocus, Walker T. Knapp, Benjamin T. Steinman, Kristina Knauss, Jonathan Stashenko, Sinead L. McWeeney, Eden Fitsum, Chloe Autry, Kristin L. Wustholz

**Affiliations:** † Department of Chemistry, 8604 William & Mary, PO Box 8795, Williamsburg, Virginia 23187-8795, United States

## Abstract

The identification of fluorescent dyes in cultural heritage materials is challenging due to analyte fading, as well as sample scarcity and complexity. Here, we demonstrate a blinking-based methodology to identify single dye molecules in ink, relying solely on the dyes’ intrinsic fluorescence intermittency. Using widefield fluorescence microscopy, change point detection, and multinomial logistic regression, we define four quantitative determination factors that provide for positive and exclusive identification among three structurally similar rhodamine dyes. This approach is then applied to wet and dry commercial ballpoint ink samples and demonstrates the presence of rhodamine B, which is validated by bulk surface-enhanced Raman scattering (SERS) measurements. As compared to SERS, blinking-based identification yields exclusive and positive identification of rhodamine dyes with single-molecule sensitivity and without the need for plasmonic substrates. This minimally invasive and ultrasensitive method offers a powerful new tool for characterizing artists’ materials, opening opportunities for conservation and heritage science.

## Graphical abstract

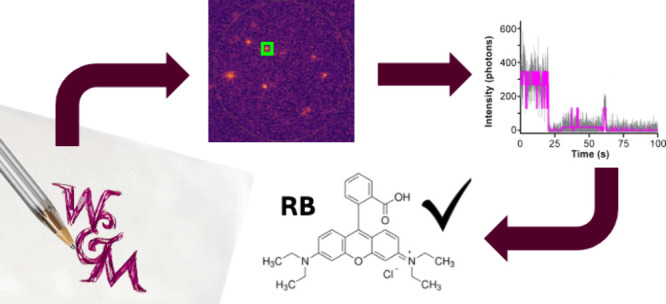

## Introduction

The analysis of natural organic colorants in historical artworks remains a major challenge in conservation science.^1–3^ These pigments are highly prone to fading, making their identification both crucial and difficult. While conventional analytical techniques such as UV/visible spectroscopy, fluorimetry, multispectral imaging, high-performance liquid chromatography (HPLC), and Raman spectroscopy have been applied to this problem, these methods lack the sensitivity and specificity needed for trace identification of organic colorants. For example, Raman spectroscopy provides molecular fingerprints but is relatively insensitive and hindered by strong background fluorescence. Surface-enhanced Raman scattering (SERS) spectroscopy can address these limitations by amplifying Raman signals through interactions with plasmonic metal nanostructures such as gold or silver nanoparticles. Adsorption to these metallic surfaces not only suppresses interfering fluorescence but can also increase sensitivity down to the level of single molecules.^4,5^ Indeed, SERS has been successfully applied to the identification of fugitive pigments and other artists’ materials in cultural heritage research.^6–9^ However, its success relies on delivering the analyte within a few nanometers of the metal surface, a significant challenge when working with unknown or compositionally complex samples that have diverse physical and chemical properties. Various strategies including sample pretreatment^10,11^ and electrochemical SERS (EC-SERS)^12,13^ have been explored to improve analyte adsorption for SERS, but these approaches add extra steps and may be unsuitable for nontargeted analysis, reduce sensitivity, or pose risks to fragile or irreplaceable art samples. There is an ongoing need for a technique that achieves single-molecule sensitivity without relying on plasmonic substrates or dealing with complex analyte–surface interactions.

Single-molecule fluorescence (SMF) spectroscopy provides sensitivity comparable to SERS while bypassing the plasmonic substrate, though at the expense of exquisite chemical selectivity afforded by vibrational spectroscopy. Traditionally, SMF involves adding *known* fluorophores to a sample to image and probe their local environments through fluorescence spectral, lifetime, polarization, or blinking measurements. Blinking is defined as the stochastic switching between fluorescent (“on”) and nonfluorescent (“off”) events that occurs for individual fluorophores under continuous laser excitation. We recently showed that this intrinsic fluorescence blinking of *unknown* molecules can be used as a barcode for their identification. Blink-based multiplexing (BBM) treats each molecule’s blinking pattern as a unique ‘fingerprint’ for identification.^14,15^ Although originally envisioned as a tool for multiplexed single-molecule or super-resolved imaging, BBM also holds promise as an analytical technique for art conservation. For example, we recently showed that organic fluorophores such as anthraquinones, BODIPYs, and rhodamines can be accurately differentiated using BBM.^16^ Here, we develop an analytical methodology to identify unknown fluorophores in artists’ materials with single-molecule sensitivity, based solely on blinking.

Prized for their bright, fluorescent colors, rhodamine dyes are widely used in artists’ materials such as pastels, daylight fluorescent paints, and inks.^6,17–20^ Their use in archival materials as well as modern and contemporary works, coupled with their tendency to fade, makes rhodamines a key target in conservation and an ideal model for developing a blinking-based identification method. In this study, we measure the blinking dynamics of three structurally related rhodamine dyes, rhodamine 123 (R123), rhodamine 6G (R6G), and rhodamine B (RB) – using widefield fluorescence microscopy. Statistical analysis and machine learning tools, such as change point detection (CPD) and multinomial logistic regression (MLR) are used to quantify blinking statistics and establish a set of quantitative determination factors that provide for positive and exclusive identification among rhodamine dyes. This protocol is then applied to wet and dry commercial ballpoint ink, complex samples that could be encountered in the conservation setting. Our results demonstrate the successful identification of RB in ballpoint ink with single-molecule sensitivity, which is confirmed by bulk SERS measurements. This work underscores the tremendous potential of blinking-based identification for identifying artists’ materials at the ultimate level of sensitivity, offering minimally invasive analysis without the added complexity or surface interactions of plasmonic substrates, a key advantage when working with limited and irreplaceable art samples.

## Results and Discussion

To transform BBM via MLR into an accurate identification tool for cultural heritage applications requires measuring the blinking dynamics of many individual molecules, establishing a set of quantitative determination factors for single-molecule identification and then testing the approach on real-world samples. We performed single-molecule imaging using a widefield fluorescence microscope equipped with an EMCCD camera. Figure 1 shows representative blinking dynamics of R123, R6G, and RB under 532 nm excitation. Consistent with prior studies, the blinking dynamics of rhodamine dyes immobilized on glass appears to be quite complex, with nonbinary fluorescence intensities and a variety of event time scales observed within each trajectory.^14,16,21^ To record and analyze blinking from individual emitters, we incorporated a change point detection (CPD) algorithm^22^ with the widely used ThunderSTORM software,^23^ allowing us to quantify a set of ten independent blinking statistics for each super-resolved emitter. The complete blinking trace processing tool is available on GitLab (https://gitlab.com/wustholz-lab/emccd-tools/thunderstorm-trace-processing). These CPD-derived statistics relate to the number, intensity, duration, and type (i.e., fluorescent “on” or nonfluorescent “off”) of events in each blinking trace. For example, the number of unique fluorescence intensity levels (*N*_*on*_), time-averaged fluorescence intensity (⟨*I*_*t*_⟩), and the average duration of fluorescence before a nonfluorescent event (⟨*t*_*on*_⟩).

**Figure 1:**
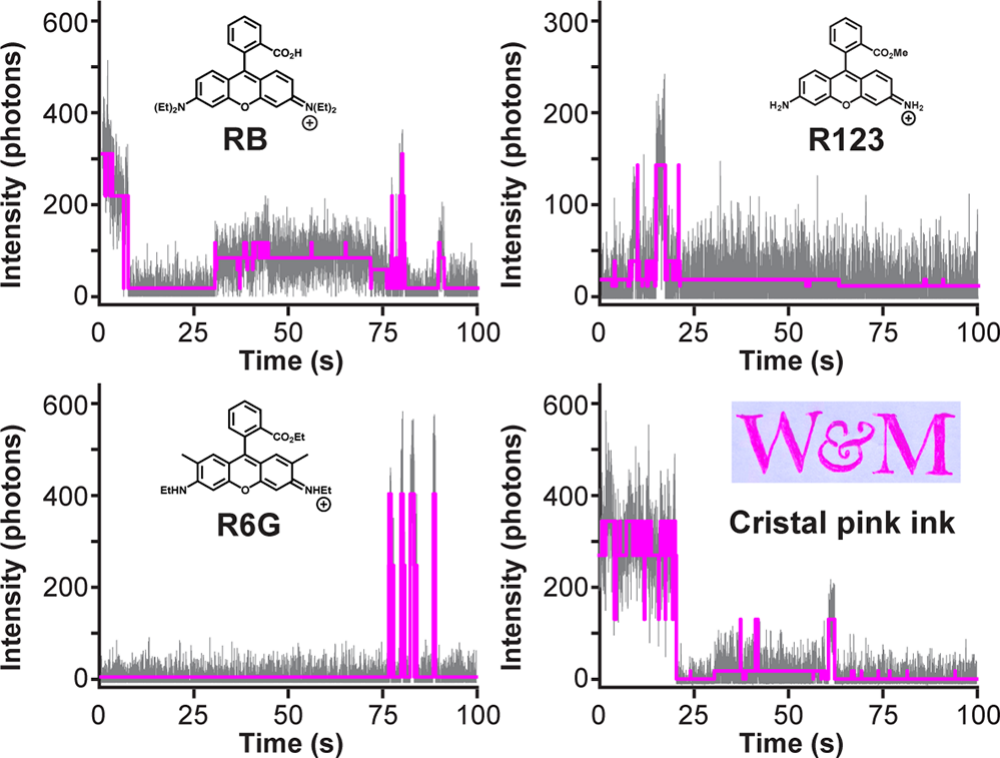
Representative blinking dynamics of RB, R123, R6G, and BIC Cristal pink ink obtained using 532 nm excitation, 1.1 kW/cm^2^ power density, 20 ms bin time and EMCCD detection, shown with (magenta line) CPD analysis.

Although a qualitative comparison of the blinking traces in Figure 1 does not show any obvious trends between R123, R6G, and RB, consistent with blinking being a stochastic process, CPD analysis reveals differences in some blinking statistics. For example, *N*_*on*_ is 36, 14, and 61 for these particular molecules of R123, R6G, and RB, respectively. ⟨*I*_*t*_⟩ changes from 22 to 23 to 69 across the same series. Significant differences in ⟨*t*_*on*_⟩ are also observed (i.e., from 5.3 s for R123 to 0.4 s for R6G to 4.6 s for RB). Similar variations in the average blinking statistics for collections of 272 R123, 117 R6G, and 177 RB molecules are observed (Table S1). For example, the average number of unique fluorescence intensity levels (⟨*N*_*on*_⟩) are 30 ± 40, 20 ± 30, and 60 ± 60 of R123, R6G, and RB, respectively, where the error corresponds to the standard deviation from the mean. Although comparing these average values begins to shed some light on which blinking statistics may be different among these structurally similar rhodamine dyes, the distributions of individual values are broad and considerably overlapped, consistent with the large standard deviations reported in Table S1. The average values of the CPD-derived blinking statistics alone cannot provide for accurate identification. To establish quantitative determination factors for identification, we implemented an approach that leverages the differences in blinking behavior between classes, as quantified using both CPD and MLR.

We previously showed that MLR, a supervised soft-classification algorithm, can provide for rapid, accurate classification of individual emitters (e.g., BODIPY versus rhodamine dyes) by using multiple blinking statistics as input predictors.^15,16^ In MLR, the probability that a molecule belongs to class A (*P*_*A*_) is modeled as a logistic (sigmoid) function with each blinking statistic contributing to the prediction via a regression coefficient:

$$P_{A}\left(x,y,...\right)={\left[1+\exp\left(-\left(\overset{n}{\underset{j}{\sum }}m_{j}x_{j}+b\right)\right)\right]}^{-1}$$

The output from this model is a set of regression coefficients, *m*_*j*_, associated with each input predictor *x*_*j*_ (e.g., *N*_*on*_, ⟨*I*_*t*_⟩, ⟨*t*_*on*_⟩, etc.) as well as an intercept (*b*), and the predicted *P*_*A*_ values, all of which might be leveraged for identification. In practical terms, the first step in this classification process is to measure the blinking dynamics of many molecules of each class (e.g., R123, R6G, and RB) and perform corresponding CPD analysis. Next, the blinking statistics for each molecule along with its class are input to the model, which yields a best-fit logistic function for each comparison (e.g., R123 versus R6G). The blinking dynamics of an unknown molecule can then be evaluated against these functions to determine its class.

To test if MLR can be used to identify rhodamine dyes in ink, we first performed a series of cross- and self-comparisons among many molecules of R123, R6G, and RB to determine sets of criteria for positive or exclusive identification. For example, if the blinking dynamics of R123 are compared to those of other molecules of R123, the MLR model should output a set of data consistent with positive identification for R123. On the other hand, if a molecule of R123 is compared to the R6G data set, the MLR model should produce values that exclude R6G as the class. What are these determination factors? One of the advantages of logistic regression as compared to other classification models is that it produces interpretable outputs (i.e., fit parameters and *P*_*A*_ values) that can be used to determine the factors that govern identification. For example, since the ten blinking statistics are normalized and uncorrelated (Figure S1), the total magnitude of the regression coefficients (∑*_j_* |*m*_*j*_|) is proportional to class differences. Therefore, for self-comparisons, denoted A/A (e.g., R123/R123, R6G/R6G, RB/RB), MLR should produce ∑*_j_* |*m*_*j*_| values close to zero. MLR cross-comparisons, denoted as A/B (e.g., R123/R6G, R123/RB, and R6G/RB), should produce larger ∑*_j_* |*m*_*j*_| values. Table 1 presents the MLR results for cross- and self-comparisons between R123, R6G, and RB. The ∑*_j_* |*m*_*j*_| values for R123/R6G, R123/RB, and R6G/RB classifications are nonzero at 5.9, 11.8, and 5.6, respectively, consistent with the model effectively distinguishing between classes. However, R123/R123, R6G/R6G, and RB/RB self-comparisons produced ∑*_j_* |*m*_*j*_| values that are closer to zero (i.e., from 1.1 to 3.2), consistent with the data being from one class (positive identification).

**Table 1:** Summary of MLR and CPD Results for Cross- and Self-Comparisons between R123, R6G, RB, and BIC Ink Samples Used to Establish Positive or Exclusive Identification^a^

	Comparison	∑ ***_j_*** | ***m*_*j*_** |	Minimum Accuracy	90% Accuracy? [Yes/No]	Average % Difference in Blinking Statistics
exclusive identification	R123/R6G	5.9	76	Yes	47.2
	R123/RB	11.8	81	Yes	39.0
	R6G/RB	5.6	76	Yes	67.2
	BIC/R123	12.2	82	Yes	50.6
	BIC/R6G	7.6	79	Yes	69.3
positive identification	R123/R123	1.8	49	No	17.5
	R6G/R6G	3.2	47	No	21.0
	RB/RB	1.4	47	No	15.1
	BIC/BIC	1.2	47	No	13.4
	BIC/RB	1.9	55	No	16.0

a Four determination factors are evaluated for each pair: the sum of the MLR coefficient magnitudes (∑ ***_j_*** | ***m*_*j*_** |), the minimum classification accuracy, whether the 90% classification accuracy threshold is reached [yes/no], and the average percent difference in CPD-derived blinking statistics between classes.

In addition to fit parameters, the MLR also produces probability outputs that can be used to make class predictions, generate a confusion matrix of predicted versus actual outcomes, and calculate classification accuracy (i.e., the percentage of correct predictions to total cases). In a binary classification model, accuracy varies from 100% (i.e., perfect classification) to ∼ 50% (i.e., the model is unable to distinguish classes and is performing no better than random chance). Therefore, classification accuracy is expected to be a useful determination factor. For cross-comparisons (A and B), where MLR should be able to effectively distinguish between classes, the model is expected to produce many accurate predictions and a high classification accuracy. However, for self-comparisons (A/A), MLR should *not* be able to distinguish classes, and the model should produce a low classification accuracy near 50%. In this framework, a low classification accuracy corresponds to positive identification.

Table 1 presents MLR accuracy metrics from cross- and self-comparisons between R123, R6G, and RB. The “minimum” classification accuracy corresponds to the case when all of the blinking data, even the molecules that yield the most uncertain classifications (i.e., corresponding to *P*_*A*_ values near 50%), are included. Consistent with prior studies,^15,16^ the minimum classification accuracies between different classes are >75%, meaning the model can effectively differentiate between R123, R6G, and RB. However, by implementing probability thresholds (i.e., *P*_*A*_ values of ∼ 80%) and discarding “less certain” data from the set, binary A/B classification accuracies of ≥ 90% are achieved between these structurally similar rhodamine dyes (Table 1 and Figure S2).^16^ Corresponding results from the self-comparisons are markedly different. When two data sets from the same class are compared, the minimum accuracies from MLR are between 47% and 55%, confirming that the model correctly treats the data as indistinguishable. Furthermore, even when probability thresholds are implemented, higher classification accuracies cannot be achieved (Figure S2). These results demonstrate that the minimum classification accuracy and whether 90% classification accuracy can be achieved via thresholding are robust determination factors. Finally, although the individual CPD-derived blinking statistics cannot alone provide for accurate identification, the average differences in these statistics between classes may offer another useful determination factor. Indeed, Table 1 shows that the average percent difference across the ten blinking statistics (i.e., $\frac{1}{10}\overset{10}{\underset{j=1}{\sum }}\left(\frac{\left|\overset{®}{x_{A,j}\;}-\overset{®}{x_{B,j}\;}\right|}{\frac{1}{2}\left(\overset{®}{x_{A,j}\;}+\overset{®}{x_{B,j}\;}\right)}\times 100\right)$ ), is substantial between classes (i.e., ranging from 39% to 69%), whereas corresponding values for self-comparisons are relatively modest (i.e., from 15% to 21%).

Taken altogether, the data in Table 1 demonstrate that CPD and MLR analyses provide a set of four determination factors for identifying rhodamine dyes based on blinking. The blinking dynamics of two rhodamine dyes is the same (positive identification) when: 1) the minimum classification accuracy is ∼ 50% (the model treats the data as indistinguishable), 2) classification accuracy of 90% cannot be reached via thresholding, 3) the value of ∑*_j_* |*m*_*j*_| is between 1.4 and 3.2, and 4) the average difference in CPD statistics is modest (i.e., 15% to 21%). Similarly, the blinking dynamics of two rhodamine dyes are different (exclusive identification) when: 1) the minimum classification accuracy is >75%, 2) classification accuracy of 90% can be reached, 3) the value of ∑*_j_* |*m*_*j*_| is ≥ 5, and 4) the average difference in CPD statistics is relatively large (i.e., 39% to 69%). Although each determination factor provides some discriminatory power, using a set of four yields a more robust and generalizable basis for identification. Furthermore, while the average percent difference factor does not require MLR analysis, these CPD statistics are quite variable across systems and conditions,^14–16^ making this factor the least reliable when used alone. Figure 2 summarizes the mean determination factors and standard deviations derived from multiple comparisons among reference dyes and the corresponding workflow for identifying unknown rhodamine dyes.

**Figure 2:**
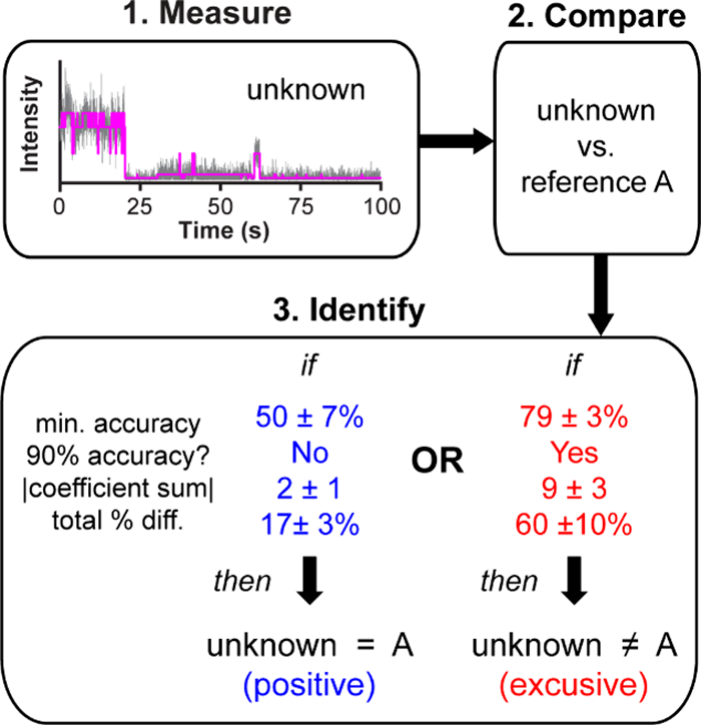
Workflow for blinking-based identification of unknown rhodamine dyes. Step 1: measure blinking dynamics of unknown molecules and perform CPD analysis. Step 2: Compare extracted blinking statistics from the unknown against a reference data set (reference A) using MLR. Step 3: Identify the unknown based on four determination factors—either assigning the unknown as matching reference A (positive) or excluding it as nonmatching (exclusive). Average determination factors are based on the data in Table 1, where the error corresponds to standard deviation from the mean.

Though the determination factors for positive versus exclusive identification shown in Figure 2 are statistically distinct, the ultimate test of these metrics is to apply them to real-world samples containing an unknown analyte. To this end, we examined wet and dry samples of colored ink, thought to contain rhodamines, that are relevant to cultural heritage research.^18–20^ Figure 1 shows a photograph of our institutional logo, drawn with a BIC Cristal pink pen, which displays intense pink coloration. Since ballpoint pen pinks are complex mixtures of dyes, pigments, and additional components like softeners and resins to improve handling properties, sample treatment strategies such as thin-layer chromatography (TLC) have been used to isolate the embedded colorant(s).^18^ Indeed, blinking measurements of untreated ink samples yielded videos that contained a few bright spots within a broad and diffuse fluorescent background, consistent with contamination from weakly fluorescent materials such as polymeric resins. Therefore, we implemented a TLC approach developed by Alyami and co-workers,^18^ to separate the colorant(s) from the ink matrix. Wet ink was obtained directly from the pen filament, while dried ink was removed from paper by using a cotton swab moistened with ethanol. TLC of these ink samples yielded a prominent pink and UV-fluorescent band at *R*_*f*_ = 0.56. The dye-doped silica gel was removed from this area and extracted into ethanol for imaging (i.e., ∼15 emitters in the imaging area).

Figure 1 shows a representative blinking trace of an individual “BIC” molecule obtained from a wet ink. The CPD-derived blinking statistics of this molecule (e.g., *N*_*on*_ = 73, ⟨*I*_*t*_⟩ = 77, and ⟨*t*_*on*_⟩ = 3.2 s) appear to differ most strongly from those of R123 and R6G, while showing the greatest similarity to RB. To unambiguously identify the analyte, we measured the blinking dynamics of 199 BIC molecules and used CPD and MLR to compare blinking to those of R123, R6G, and RB reference dyes. Table 1 shows that when BIC is compared to R123 and R6G, all four identification factors fall soundly within the exclusive identification range: high classification accuracies, low MLR coefficients, and significant differences in blinking statistics. On the other hand, comparison of BIC to RB shows that all four determination factors are consistent with positive identification for RB (i.e., classification accuracy is 55%, 90% accuracy cannot be reached, ∑*_j_* |*m*_*j*_| is 1.9, and differences in CPD statistics are 16%). Taken together, CPD and MLR analyses of wet BIC ink exclude R123 and R6G as the analyte and positively identify BIC as RB.

For blinking measurements of dry ink samples, three determination factors fall within the positive identification range for RB as listed in Figure 2 (i.e., 90% accuracy cannot be reached, ∑*_j_* |*m*_*j*_| is 2.9, and differences in CPD statistics are 16%). The dried BIC ink samples exhibit a slightly higher minimum classification of 63%, which may be due to minor contamination introduced during cotton swab extraction. This comparison indicates that the range of acceptable minimum accuracies or other determination factors in Figure 2 may increase with further testing of additional dyes, conditions, treatments, and complex real-world samples. All four determination factors exclude R123 and R6G as the analyte (i.e., dry BIC/R123 and dry BIC/R6G yielded minimum accuracies of 86% and 83%, with 90% accuracy achieved, ∑*_j_* |*m*_*j*_| values of 12.5 and 8.8, and differences in CPD statistics of 51% and 69%, respectively). For completeness, we also compared blinking from wet and dry ink samples and found that they could not be distinguished using MLR.

As a final test of this blinking-based identification approach, we performed a separate SERS analysis of the ink. Figure 3 shows the SERS spectra of the TLC-separated BIC ink as compared to RB and the enhancing substrate, citrate-reduced silver nanoparticles (AgNPs). Major and distinguishing SERS peaks are observed at 1521 cm^–1^, 1506 cm^–1^, 1276 cm^–1^ 1194 cm^–1^, 789 cm^–1^, 731 cm^–1^, 621 cm^–1^, and 485 cm^–1^, in excellent agreement with RB.^18^ Moreover, prominent SERS peaks for R123 and R6G are notably absent (e.g., 1587 cm, 610 cm^–1^, and 485 cm^–1^ for R123 and 600 cm^–1^ for R6G).^4,24^ Collectively, SERS analysis confirms the blinking-based identification of RB as the analyte.

**Figure 3:**
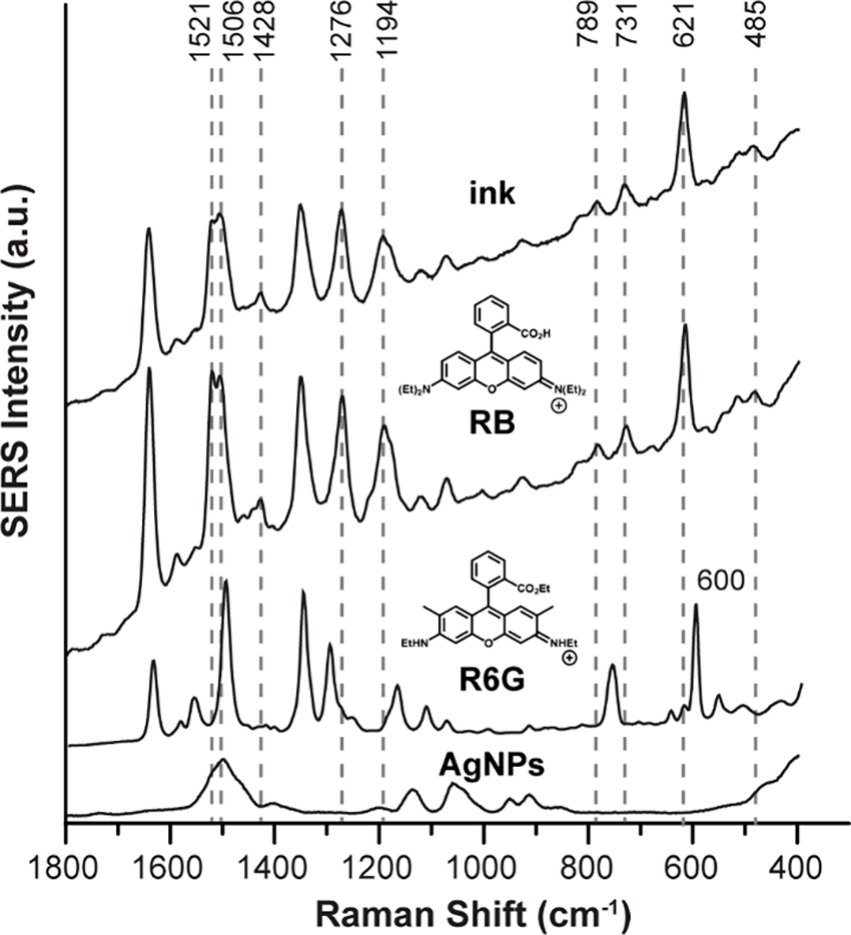
SERS spectra of the TLC-separated BIC ink as compared to RB, R6G, and AgNPs obtained at 632.8 nm excitation.

Blinking-based identification offers a powerful new approach for characterizing fluorescent dyes in cultural heritage materials. This study demonstrates that exceptionally small samples, such as microscopic solids judiciously removed from an object or colored extracts from ink on paper, are adequate to provide for positive identification of single rhodamine molecules, enabling discrimination among structurally similar chromophores without the need for SERS or plasmonic substrates. For both wet and dry ink samples, the four determination factors outlined in Figure 2 reliably exclude candidate dyes and confirm the analyte. While TLC remains an essential pretreatment step for modern ink samples due to their high chemical complexity and autofluorescence, ongoing work is focused on developing simpler extraction protocols for dyes, pigments, and paint samples. These results highlight the importance of mechanistic studies of blinking in complex environments as well as development of reference blinking libraries from modern and aged artists’ materials. Future efforts will extend this blinking-based identification to additional dye classes such as anthraquinones, which are prevalent in cultural heritage materials and known to undergo a different blinking mechanism.^16,25^ By analyzing complex mixtures of such dyes, which can be present in art from biological sources and/or artists’ choices, this approach holds the potential to reveal the full fluorophore distribution in cultural heritage objects, information otherwise hidden by ensemble averaging.

## Supplementary Material



## Notes

Experimental details, average CPD statistics, additional statistical analyses, complete fitting results (PDF)
