# Supplementary material for: Blinking-Based Identification of Single Dye Molecules in Ink
Source: Anal Chem. 2026 Jan 2;98(1):63–8. doi: 10.1021/acs.analchem.5c06137 (PMC12809631; doi:10.1021/acs.analchem.5c06137)
Supplement: Supplementary file 1 [file ac5c06137_si_001.pdf]

Supporting Information

Blinking-Based Identification of Single Dye Molecules in Ink

Alisha J. Khodabocus,[a] Walker T. Knapp,[a] Benjamin T. Steinman,[a] Kristina Knauss,[a] Jonathan Stashenko,[a] Sinead L. McWeeney,[a] Eden Fitsum,[a] Chloe Autry,[a] Kristin L. Wustholz [a]*

[a]Department of Chemistry, William & Mary, PO Box 8795, Williamsburg, VA 23187-8795

*Corresponding author: Prof. Kristin Wustholz, Department of Chemistry, William & Mart, PO Box 8795, Williamsburg, VA, USA. Tel: (757) 221-2675, Email: kwustholz@wm.edu

Contents

- **Experimental details** (S2-S5) including complete descriptions of materials (S2), single-molecule imaging and analysis (S2-S4), and SERS measurements (S4-S5)
- **Table S1.** Average CPD-derived blinking statistics of all samples included in this study (S6)
- **Figure S1**. False-colored Pearson correlation coefficient plot of the 10 blinking statistics used in this study (S7)
- **Figure S2.** Plots of classification accuracy and data retention as the classification threshold is increased (S8)
- **Table S2.** Best-fit parameters for MLR classification (S9)
- **References** (S10)

Experimental Details

Materials

High purity (99%) R123, R6G, and RB were used as received from Acros Organics. Solutions of RB, R6G, and R123 were prepared in ethanol (Pharmco, 200-proof) and sonicated for ~20 s prior to use. Ultrapure water (Millipore Sigma Synergy, 18.2 MΩ cm) was used to clean glassware, perform thin-layer chromatography (TLC), and synthesize the SERS substrate. BIC Cristal Pink™ ballpoint pens were sourced commercially.

Single-Molecule Imaging & Blinking Analysis

Samples for single-molecule blinking measurements of reference dyes were prepared by spin coating 35 μL of 10 nM dye solution onto a clean glass coverslip using a spin coater (Laurell Technologies, WS-650Mz-23NPPB) operating at 3000 rpm for 30 s. Wet ink from the filament of a BIC Cristal Pink pen was placed into a clean glass vial. About 3 mg of ink was put into 30 μL of ethanol. Consistent with a prior study of BIC inks,[1] thin-layer chromatography (TLC) was performed on the resulting ink solution using silica gel plates (Fluka Analytical, 5 μm, aluminum backing), by spotting ~1 μL of ink solution and 10^{-4} M RB solution in separate lanes with a mobile phase of 7:3.5:3 v/v/v ethyl acetate:ethanol:water.[2] After 10 min of development and subsequent drying, a ~0.7 g sample of dye-doped silica gel corresponding to the pink and UV-fluorescent band at an R_f value of 0.56 was removed from the plate using surgical scalpel (Feather Safety Razor Company) and then extracted in 500 μL of ethanol overnight. To remove residual silica gel, the extracted solution was centrifuged for 5 min at 9000 rpm. Finally, 10 μL of the resulting supernatant was diluted with 1000 μL of ethanol for blinking measurements, resulting in a density of ~15 emitters across the 46 μm^2 imaging area. Corresponding samples of dry ink were performed by applying the ink to a 2×2 cm^2 area of standard printer paper, which was allowed to dry overnight, and then extracted by gentle swabbing with an ethanol-soaked cotton swab (Fisher, REF-22363172). The resulting pale pink swab was soaked in 500 μL ethanol for 24h before undergoing TLC as above.

To the resulting reference dye and ink samples were added a small amount of nanoscale beads (Spherotech, FP-0270-2, 0.26 μm diameter) as fiducial markers, to facilitate microscope alignment and perform drift corrections in post-processing. For this step, 15 μL of a 1% w/v aqueous suspension of beads that had been sonicated for 15 s was pipetted directly onto the coverslip,

allowed to sit for 5 min, and then further dried by spinning at 500 rpm for 60 s. The resulting samples containing dye molecules and fiducial markers were fastened in a custom aluminum flow cell, flushed with dry nitrogen throughout the experiment, and placed on a widefield microscope (Nikon, TiU) coupled to a 532 nm laser (Spectra Physics, Excelsior) operating at 1.11 kW cm^{-2} power density. The beam was directed through a 100× oil-immersion objective (Nikon Plan Fluor, NA = 1.3). Emitted fluorescence passed through a long-pass filter (Semrock, LP03-532RS-2S) before collection via an EMCCD camera (ANDOR iXon Ultra & Life 897). Widefield image sequences were recorded for 100 s using a 20-ms integration time and gain setting of 200 under cw laser excitation.

To extract blinking data (i.e., emission-time trajectories) from each video, we employed an implementation of ThunderSTORM[3] (i.e., b-spline wavelet filter analysis, with order and scale set to 4 and 3, respectively) created by Izeddin and coworkers[4] to approximate the locations of molecules within each frame. A complete list of sub-diffraction localized detections was then determined via ThunderSTORM's maximum likelihood estimate approach with a fit radius set to 192 nm. Fiducial-based drift correction was used to normalize the locations of each detection. Because the samples in this study are relatively dilute (averaging 10–20 molecules per video), a distance-based merging protocol was used to produce a de-duplicated list of molecules, grouping detections within ~150 nm as originating from the same molecule. A custom plugin to export these detections in csv format was made and is published under the GPLv3.0 License (https://gitlab.com/wustholz-lab/emccd-tools/thunderstorm-merged-export). Custom post-processing using pandas[5] is then applied to filter out extraneous emitters, defined as those appearing in fewer than 10 frames or with an intensity less than 6 standard deviations above the noise. For each remaining molecule, the average location, point-spread-function (PSF) width, and noise floor across all detections were determined. Finally, a single Gaussian PSF constructed from these values was fit to the original data on each frame to determine intensity trajectories, where intensity corresponds to the integrated area under the PSF.

To quantify the blinking dynamics of each molecule, we applied change point detection (CPD) analysis using a modified version of the algorithm developed by Kopera and coworkers.[6] A generalized likelihood ratio was first used to identify times at which the signal is likely to have changed, following the approach of Watkins and Yang.[7] The segments between these change points are then clustered into states using a mixture model that assumes each segment's measured

intensity is drawn from a Poisson-Gamma-Normal (PGN) distribution, in contrast to Kopera et al., which assumed a normal distribution. This modification better captures the characteristics of EMCCD measurements, particularly the readout noise inherent to this detector class.[8] The set of merged segments generated by the PGN mixture model is then used to determine blinking statistics within a binary intensity framework, where "on" events are those with intensities one standard deviation above lowest-intensity segment and all others events are "off". For this work, 10 CPD-derived blinking statistics were determined: the number of distinct emission intensities (N_I), minimum and maximum intensities (I_{min} and I_{max}), time-averaged emission intensity ($\langle I \rangle_t$), average duration of on and off segments ($\langle t_{on,seg} \rangle$ and $\langle t_{off,seg} \rangle$), average duration of successive on and off segments, called "intervals" ($\langle t_{on,int} \rangle$ and $\langle t_{off,int} \rangle$), and the number of on and off segments per 100 s observation period (N_{on} and N_{off}). This implementation of CPD for EMCCD data, which was written in Python 3.10 and relied heavily upon Numba[9] for performance acceleration, was published on GitLab (https://gitlab.com/wustholz-lab/pycpd).

CPD statistics were standardized by z-score normalization and then used as input predictors, along with molecule identity, for fitting and classification using multinomial logistic regression (MLR) analysis (Matlab R2023b, mnrfit and mnrval).[10] After the fit parameters are established using MLR, molecule identities are removed, the dataset is split into training (90%) and testing (10%) sets, and 10-fold cross validation is performed to assess the model's predictions when emitter class is unknown. The MLR classifications of binary self- and cross-comparisons were performed in triplicate using randomly-selected and equivalently-sized subsets of the data, which produced consistent results. Several alternative supervised learning algorithms (e.g., K-nearest neighbors classifier (KNC), linear support vector classifier (LSVC), Kernel SVC (KSVC), Random forest) were tested in initial control experiments involving R6G and RB. Since all models produced comparable classification accuracies under 10-fold cross-validation, MLR was selected because it provides well-behaved and interpretable class probabilities.

SERS Measurements

Citrate-reduced silver nanoparticles (AgNPs) were made for SERS studies of samples of solid dyes and commercial ballpoint ink. Synthesis glassware was cleaned with aqua regia and rinsed thoroughly with ultrapure water (ThermoScientific, EasyPure II, 18.2 MΩ cm) prior to use. Silver nitrate (Strem Chemicals, 99.9995%) and sodium citrate trihydrate (Sigma Aldrich) were used to

make AgNPs according to the Lee-Meisel method,[11] resulting in an opaque, gray-green colloidal suspension. The resulting AgNPs were centrifuged (Eppendorf, MiniSpin, 1-mL aliquots with ~0.97 mL supernatant removed) for three cycles at a relative centrifugal force of ~5,400 g at 15 min per cycle, to yield ~0.3 mL of concentrated AgNPs. Microscopic samples of solid dyes were placed on clean glass coverslips and coated in 2 μL AgNPs for SERS measurements. For corresponding measurements of ink, we performed TLC-SERS as previously reported by Alyami and coworkers.[2] Briefly, the filament of a ballpoint pen was removed and ~1 μL droplet of ink was directly deposited on a TLC plate. For TLC-SERS, plates were developed using ethyl acetate (Fisher Scientific) for ~1 h to yield a single pink, fluorescent spot ($R_f \approx 0.4$). Approximately 2 μL of AgNP paste was applied directly to the pink spot on the TLC plate and allowed to dry for 24 h. The next day, the ~4 mm^2 AgNP-coated spot was scraped from the TLC plate using a surgical scalpel and placed atop a clean glass coverslip for SERS analysis.

SERS measurements were performed on a homebuilt inverted microscope (Nikon, TiU) equipped with a spectrograph (Princeton Instruments, SP2356), CCD camera (Princeton Instruments, PIXIS:100B-Excelon), HeNe laser (Research Electro-Optics, LHRP-1701), and 20× objective (Nikon CFI, N.A. = 0.5) as described previously. Raman scattering was dispersed using a 600 g/mm grating blazed at 500 nm and calibrated using a cyclohexane standard. Typical excitation power and acquisition time were set to ~20 μW and 30 s, respectively, to maximize signal while preventing molecular photobleaching. SERS spectral analysis was performed in OriginPro 2023 software.

Table S1. Average CPD-derived blinking statistics measured using single-molecule imaging for R123 (n = 272), R6G (n = 117), RB (n = 177), and molecules extracted from BIC Cristal Pink ink (n = 199) immobilized on glass. Errors correspond to the standard deviation from the mean. The intensities have units of counts per 20-ms bin time and all durations are in seconds.

**Sample**	N_I	N_{on}	N_{off}	I_{min}	I_{max}	$\langle I \rangle_t$	$\langle t_{on,seg} \rangle$	$\langle t_{off,seg} \rangle$	$\langle t_{on,int} \rangle$	$\langle t_{off,int} \rangle$
R123	4 ± 2	30 ± 40	10 ± 20	20 ± 40	160 ± 100	20 ± 60	1 ± 1	4 ± 5	10 ± 20	4 ± 9
R6G	3 ± 2	20 ± 30	10 ± 10	10 ± 10	250 ± 300	30 ± 30	0.4 ± 0.5	2 ± 4	2 ± 5	2 ± 5
RB	5 ± 2	60 ± 60	10 ± 20	20 ± 20	300 ± 300	70 ± 70	2 ± 3	3 ± 7	10 ± 10	3 ± 8
ink	5 ± 2	100 ± 100	20 ± 20	20 ± 20	300 ± 300	90 ± 80	2 ± 3	3 ± 7	10 ± 10	2 ± 5

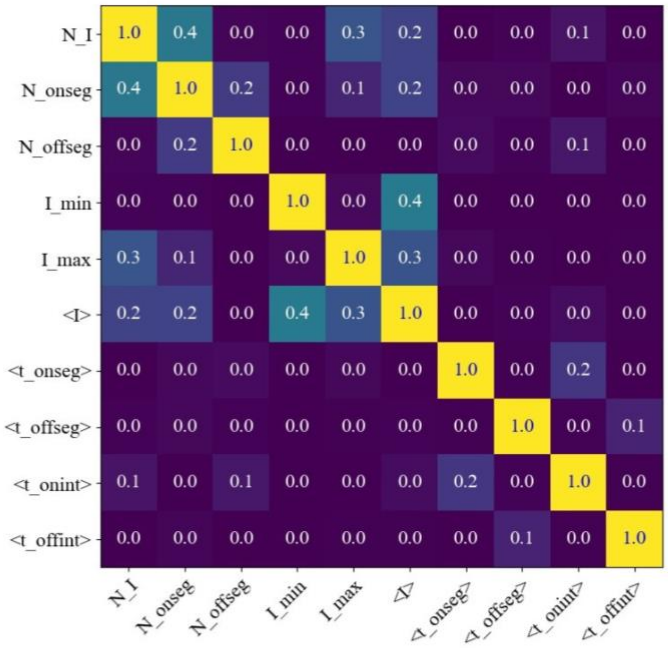

Figure S1. Pairwise Pearson R^2values between the 10 blinking statistics used in this study N_I, N_{on}, N_{off}, I_{min}, I_{max}, $\langle I \rangle_t$, $\langle t_{on,seg} \rangle$, $\langle t_{off,seg} \rangle$, $\langle t_{on,int} \rangle$, $\langle t_{off,int} \rangle$) ranged from 0 to 0.4, demonstrating weak linear correlations and supporting the interpretation that these predictors provide for largely independent information for the logistic regression model. The matrix is shown in false color, with dark blue representing $R^2 = 0$ and yellow representing $R^2 = 1$.

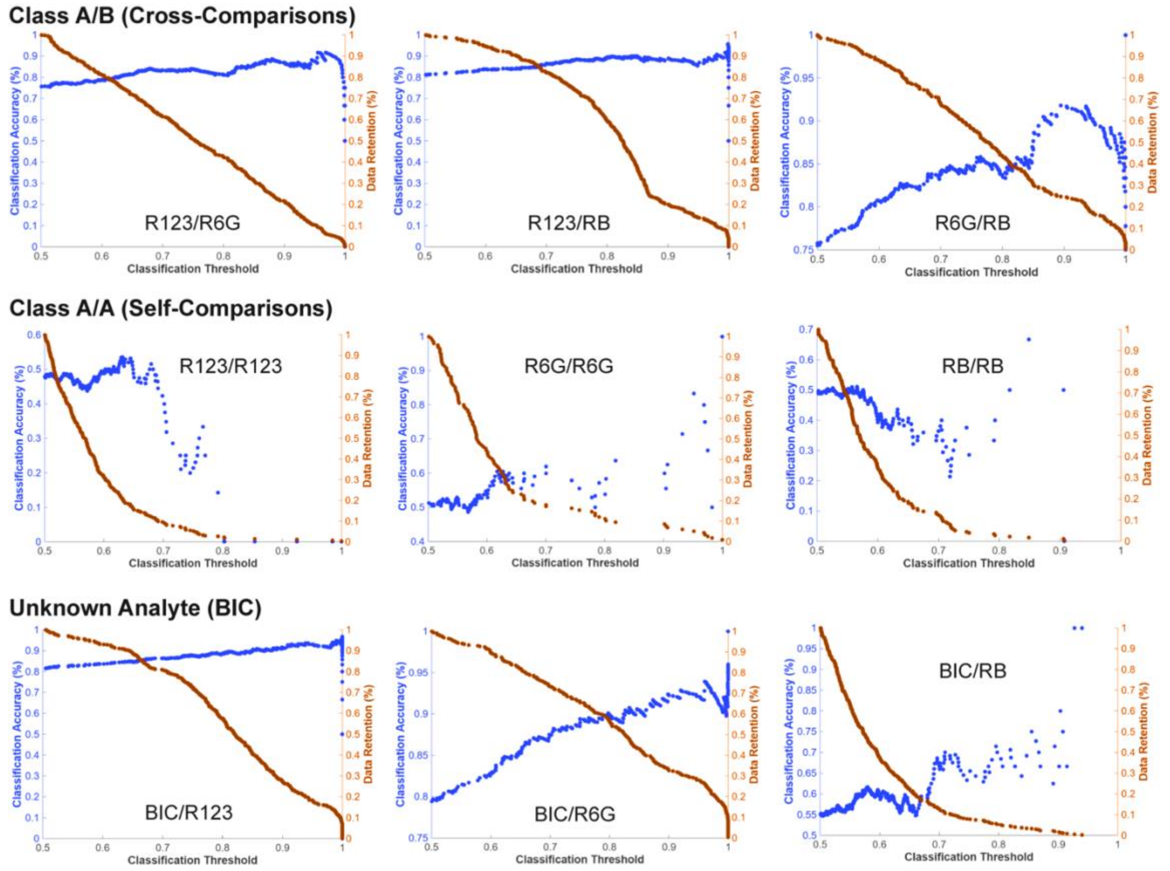

Figure S2. Plots of (blue) classification accuracy and (red) corresponding data retention (i.e., the percentage of the original dataset retained once the threshold is applied) versus classification threshold for various binary MLR-based classifications: (top) class A/B, (middle) class A/A, and (bottom) unknown analyte in BIC ink versus R123, R6G, and RB reference dyes. Points corresponding to data retention of <10% are not considered. Application of a classification threshold improves the MLR-based accuracy to ≥90% when the classes are different. Classification accuracies are plateaued at ~50% for the self-comparisons, even when a classification threshold is applied and corresponding data retention drops precipitously. The classification accuracies for BIC/R123 and BIC/R6G begin at minimum values of ~80% and improve to ≥90% with thresholding, consistent with R123 and R6G being excluded as the analyte. On the other hand, the BIC/RB minimum classification accuracy is relatively plateaued at ~55%, with only a modest improvement to ~60% with ~20% data retention. Collectively, these data are consistent with positive identification of RB in BIC.

Table S2. Best-fit parameters (i.e., regression coefficients and intercept (b)) of the 10 CPD-derived blinking statistics and associated fluorophore identity to sigmoid functions resulting from MLR-based classification between R123, R6G, RB, and BIC Cristal Pink ink.

	b	N_I	N_{on}	N_{off}	I_{min}	I_{max}	$\langle I \rangle_t$	$\langle t_{on,seg} \rangle$	$\langle t_{off,seg} \rangle$	$\langle t_{on,int} \rangle$	$\langle t_{off,int} \rangle$
R123/ R6G	1.1	0.7	0.1	0.3	1.2	-0.7	-1.2	0.4	0.4	0.5	0.4
R123/ RB	-0.1	0.3	0.3	-0.2	3.6	-0.7	-5.5	-0.7	-0.1	0.5	0.02
R6G/ RB	-1.1	-0.2	-0.4	-0.2	0.3	-0.1	-1.7	-2.0	-0.4	-0.2	-0.2
R123/ R123	0.1	-0.1	-0.2	-0.05	1.3	0.1	0.7	0.3	0.06	-0.04	-0.3
R6G/ R6G	0.0	-0.5	-0.2	0.2	-0.4	-0.2	0.9	-0.1	0.1	0.0	0.0
RB/ RB	-0.01	0.4	-0.1	0.01	0.006	0.1	0.08	0.5	0.04	-0.4	-0.2
BIC/ BIC	0.0	0.2	0.2	-0.3	-0.1	-0.2	-0.2	0.0	-0.1	-0.3	0.1
BIC/ R123	-0.1	0.3	0.3	-0.2	3.6	-0.7	-5.5	-0.7	-0.1	0.5	0.0
BIC/ R6G	1.7	-0.1	0.8	0.5	-0.5	-0.4	2.7	1.5	0.5	0.5	0.1
BIC/ RB	0.1	-0.5	0.7	-0.1	-0.1	-0.1	0.2	0.0	0.0	0.1	0.0

References

(1) Futamata, M.; Yu, Y. Y.; Yanatori, T.; Kokubun, T. Closely Adjacent Ag Nanoparticles Formed by Cationic Dyes in Solution Generating Enormous SERS Enhancement. *J. Phys. Chem. C* 2010, *114* (16), 7502–7508. https://doi.org/10.1021/jp9113877.

(2) Alyami, A.; Barton, K.; Lewis, L.; Mirabile, A.; Iacopino, D. Identification of Dye Content in Colored BIC Ballpoint Pen Inks by Raman Spectroscopy and Surface-Enhanced Raman Scattering. *J. Raman Spec.* 2019, *50* (1), 115–126. https://doi.org/10.1002/jrs.5512.

(3) Ovesný, M.; Křížek, P.; Borkovec, J.; Švindrych, Z.; Hagen, G. M. ThunderSTORM: A Comprehensive ImageJ Plug-in for PALM and STORM Data Analysis and Super-Resolution Imaging. *Bioinformatics* 2014, *30* (16), 2389–2390. https://doi.org/10.1093/bioinformatics/btu202.

(4) Izeddin, I.; Boulanger, J.; Racine, V.; Specht, C. G.; Kechkar, A.; Nair, D.; Triller, A.; Choquet, D.; Dahan, M.; Sibarita, J. B. Wavelet Analysis for Single Molecule Localization Microscopy. *Opt. Express* 2012, *20* (3), 2081. https://doi.org/10.1364/oe.20.002081.

(5) McKinney, W. Data Structures for Statistical Computing in Python. *Proceedings of the 9th Python in Science Conference* 2010, *1* (Scipy), 56–61. https://doi.org/10.25080/majora-92bf1922-00a.

(6) Kopera, K. M.; Tuckman, H. G.; Hoy, G. R.; Wustholz, K. L. Origin of Kinetic Dispersion in Eosin-Sensitized TiO2: Insights from Single-Molecule Spectroscopy. *J. Phys. Chem. C* 2021, *125* (43), 23634–23645. https://doi.org/10.1021/acs.jpcc.1c07597.

(7) Watkins, L. P.; Yang, H. Detection of Intensity Change Points in Time-Resolved Single-Molecule Measurements. *J. Phys. Chem. B* 2005, *109* (1), 617–628. https://doi.org/10.1021/jp0467548.

(8) Ryan, D. P.; Dunlap, M. K.; Gelfand, M. P.; Werner, J. H.; Van Orden, A. K.; Goodwin, P. M. A Gain Series Method for Accurate EMCCD Calibration. *Sci. Rep.* 2021, *11* (1), 1–10. https://doi.org/10.1038/s41598-021-97759-6.

(9) Lam, S. K.; Pitrou, A.; Seibert, S. Numba: A LLVM-Based Python JIT Compiler. *Proceedings of LLVM-HPC 2015: 2nd Workshop on the LLVM Compiler Infrastructure in HPC - Held in conjunction with SC 2015: The International Conference for High Performance Computing, Networking, Storage and Analysis* 2015, *2015-Janua*. https://doi.org/10.1145/2833157.2833162.

(10) Hoy, G. R.; DeSalvo, G. A.; Haile, S. H.; Smith, E. N.; Wustholz, K. L. Rapid, Accurate Classification of Single Emitters in Various Conditions and Environments for Blinking-Based Multiplexing. *J. Phys. Chem. A* 2023, *127* (15), 3518–3525. https://doi.org/10.1021/acs.jpca.3c00917.

(11) Lee, P. C.; Meisel, D. Adsorption and Surface-Enhanced Raman of Dyes on Silver and Gold Sols. *J. Phys. Chem.* 1982, *86* (17), 3391–3395. https://doi.org/10.1021/j100214a025.
